# Safety of discontinuing cotrimoxazole prophylaxis among HIV infected adults on anti-retroviral therapy in Uganda (COSTOP trial): Design

**DOI:** 10.1016/j.cct.2015.05.015

**Published:** 2015-07

**Authors:** Zacchaeus Anywaine, Andrew Abaasa, Jonathan Levin, Ronnie Kasirye, Anatoli Kamali, Heiner Grosskurth, Paula Munderi, Andrew Nunn

**Affiliations:** a MRC/UVRI Uganda Research Unit on AIDS, Entebbe, Uganda; b MRC Clinical Trials Unit at University College London, UK; c London School of Hygiene and Tropical Medicine, London, UK; d School of Public Health, Faculty of Health Sciences, University of the Witwatersrand, Johannesburg, South Africa

**Keywords:** HIV infection, Cotrimoxazole prophylaxis, Stopping cotrimoxazole, Antiretroviral treatment, Cotrimoxazole cessation study design, Uganda

## Abstract

**Introduction:**

Cotrimoxazole (CTX) prophylaxis is recommended by the World Health Organisation for HIV infected persons. However, once HIV infected patients have commenced ART in resource limited settings, the benefits of continued CTX prophylaxis are not known. The few studies that investigated the safety of discontinuing CTX prophylaxis in these settings had limitations due to their design.

**Materials and methods:**

COSTOP is a randomised double blind placebo controlled non-inferiority trial among HIV infected Ugandan adults stabilised on anti-retroviral treatment (ART). Participants with CD4 count of 250 or more cells/mm^3^ are randomised to two arms: the intervention arm in which CTX is discontinued and the control arm in which CTX prophylaxis is continued. The study aims to assess whether the intervention regimen is not inferior, with respect to the incidence of pre-defined CTX-preventable events, to the control regimen and superior with respect to the incidence of haematological adverse events.

**Discussion:**

Studies that have previously evaluated the safety of discontinuing CTX prophylaxis among HIV infected adults in resource limited settings have provided moderate to low quality evidence owing in part to methodological limitations. COSTOP is designed and conducted with sufficient rigour to answer this question. The results of the trial will assist in guiding policy recommendations.

**Conclusion:**

This paper describes the design and methodological considerations important for the conduct of CTX cessation studies.

## 1 Introduction

The use of cotrimoxazole (CTX) as prophylaxis against opportunistic infections among HIV-infected persons is part of the standard of care recommended by the World Health Organisation (WHO) [1,2]. In resource limited settings, once HIV infected patients have commenced ART, the benefits of continued prophylactic CTX medication are not known [1].

A few studies in resource limited settings [3–6] have investigated the effect of providing prophylactic CTX *versus* no CTX among patients concurrently taking ART. All these studies had limitations in that either they were observational [3], had small sample sizes [4,5] or followed participants for short periods as reviewed by Suthar et al. [6]. This systematic review also concluded that “cotrimoxazole significantly increased survival in HIV infected adults on ART. Further research is needed to determine the optimum duration of CTX treatment in these patients”. Campbell and colleagues carried out a trial in a home based care setting in rural Eastern Uganda in which 836 patients who had been on ART for a median time of 3.7 years and who had a CD4 count above 200 cells/μl were randomised at household level to continue or discontinue CTX prophylaxis in an open label design [7]. The trial was stopped at the recommendation of the DSMB following the occurrence of significantly higher rates of asymptomatic and symptomatic malaria in the group which stopped CTX (RR = 27.7, 95% CI 6.8, 113.1, p < 0.001). There was also a significantly higher rate of self-reported diarrhoea, but no difference between the two arms in the incidence of AIDS-related opportunistic infections and no deaths were reported. Recently, a study conducted in Kisumu—Kenya compared the effect of CTX cessation *versus* continuation on a composite outcome of death, malaria, pneumonia and diarrhoea among HIV infected adults stabilised on ART [8]. None of these studies used a double-blind placebo controlled design.

A WHO Guideline Development Group on CTX Prophylaxis convened in 2013 recommended the continuation of CTX prophylaxis among patients stable on ART in settings with severe bacterial infections and high malaria prevalence; but that these guidelines should be adapted to ‘national context’ [2]. There is still uncertainty within resource limited settings and further research is needed to provide evidence based recommendations for or against stopping CTX. Garnering high quality evidence requires studies with robust designs, methodological and ethical considerations.

In this paper we present the design and methods used in the conduct of the CTX prophylaxis cessation trial among HIV infected adults on ART in Uganda (trial registration number: ISRCTN44723643).

## 2 Materials and methods

COSTOP is a randomised, double-blind, placebo controlled non-inferiority trial among HIV-infected adults in Uganda that have been immunologically stabilised on ART. The objective of the study is to assess whether, in patients with CD4 count of ≥ 250 cells/mm^3^, a regime in which CTX prophylaxis is discontinued is:

(a) not inferior, with respect to the incidence of pre-defined CTX-preventable events to the control regimen in which prophylaxis with CTX is continued and
(b) superior to continuing CTX prophylaxis with respect to reducing the incidence of haematological adverse events.

The pre-defined CTX-preventable events (Table 1) are a subset of the WHO-staging events, namely those that are deemed to be CTX-preventable *a priori*.

### 2.1 Study setting and population

The study is being conducted in Uganda at the MRC/UVRI Unit clinics in Entebbe and Masaka. Patients on long-term CTX and ART care are recruited from local HIV treatment centres situated near-by.

The eligibility criteria used are as follows:

Inclusion criteria
  ❖ HIV-infected patient with documented intake of CTX for at least 6 months;
  ❖ age of ≥ 18 years;
  ❖ documented intake of ART for at least 6 months;
  ❖ clinically asymptomatic;
  ❖ 2 CD4 counts (not more than 6 months apart) of ≥ 250 cells/mm^3^, the most recent no more than 4 weeks prior to enrolment; and
  ❖ able to attend study clinics at 3-monthly intervals and in the event of intercurrent illness.
Exclusion criteria
  ❖ acute illness (opportunistic infection or other co-morbidity);
  ❖ first trimester pregnancy;
  ❖ known hypersensitivity to cotrimoxazole; and
  ❖ grade 3/4 anaemia, neutropenia or thrombocytopenia.

### 2.2 Ethical approval

Ethical permission was obtained from the Uganda Virus Research Institute Research and Ethics Committee (UVRI REC), the Uganda National Council for Science and Technology (UNCST) and the Ugandan National Drug Regulatory Authority (NDA). The trial is monitored by an Independent Data Monitoring Committee (IDMC).

### 2.3 Intervention

All participants are required to stop their regular CTX after which they are randomised to receive CTX tablets of 960 mg or a matching placebo tablet. All participants continue to receive ART from their routine providers. Trial medication is dispensed monthly for the first three months and three-monthly thereafter with a fixed number of extra tablets to allow for the possibility of late attendance. Participants are requested to return their trial medication packs with any unused tablets at scheduled clinic visits. Allocated trial treatment is discontinued in the event of the following: confirmed CD4 count drop to below 250 cells/mm^3^, participants' consent withdrawal and intercurrent illness preventing further treatment with trial drug. No additional participants are recruited to replace those withdrawn. Participants withdrawn from trial treatment due to a confirmed CD4 count drop to below 250 cells/mm^3^ or due to consent withdrawal are prescribed open label CTX. Follow-up of participants withdrawn from the study intervention continue unless the participant explicitly withdraws consent for follow-up.

### 2.4 Study schedule

A summary of the study schedule of visits and procedures is shown in Table 2. Participants are informed about the trial and provide informed consent before screening by signing the informed consent form. Illiterate participants sign by thumbprint in the presence of an independent literate witness.

At screening, potential participants are assessed for eligibility, socio-demographic and behavioural characteristics, and for their medical history (including ART use and past WHO clinical stage events). A clinical examination is conducted and Laboratory investigations include a full blood count, malaria slide and CD4 count.

The enrolment visit takes place within 2 to 4 weeks of screening; eligibility is confirmed and consent obtained for randomisation into the trial. At enrolment and each follow-up visit, routine trial procedures are performed as indicated in Table 2. Participants are seen monthly for the first three months and 3 monthly thereafter. All participants are provided with an insecticide treated mosquito net (ITN) and educated about the importance of using it. Other medications and investigations are provided as required for the management of the participant's reported disease condition. Participants are encouraged to report to the study clinics whenever they fall sick. All adverse events (AE) are assessed and appropriate management provided. Serious adverse events (SAEs) are reported to the UVRI REC, UNCST and NDA.

### 2.5 Randomization and blinding

Participants are randomised in a ratio of 1:1 to stop or continue CTX prophylaxis. The randomisation schedule was produced by an independent statistician at the MRC/UVRI Unit using random permuted blocks of variable size with separate randomisations carried out in four strata, defined by the four possible combinations of study site (Entebbe or Masaka) and baseline CD4 count (250–499 cells/mm^3^ or ≥ 500 cells/mm^3^). Neither study staff nor the endpoint review committee (ERC) members have access to the randomisation schedule. Eligible participants details are entered by the study clinician on the next available row of the enrolment register in accordance with their CD4 count. The trial number corresponding to that row is used on all trial documents and to identify the pre-labelled study medication.

### 2.6 Unblinding procedures

The randomisation codes are maintained by the independent trial statistician and a copy is held by the trial pharmacist. Unblinding is discouraged during treatment; if however, a trial clinician considers it necessary for a participant's allocated treatment to be unblinded this is first discussed with the chief investigator. If unblinding is considered appropriate the reason for unblinding is recorded in the unblinding register and an unblinding form is completed and sent to the independent statistician, or in his absence to the pharmacist. The unblinding information is disclosed to the attending clinician and is kept confidential to other study staff and is entered on the database.

### 2.7 Outcomes

There are two co-primary outcome measures: time to the first CTX preventable event (either one of the WHO staging events in Table 1 or else a death adjudicated by the ERC to be CTX preventable), and time to the first grade 3 or 4 haematological adverse event. Secondary outcome measures include the following: all-cause mortality; incidence of all CTX preventable events, all clinical events and SAEs; incidence, severity and outcome of all malaria episodes (asymptomatic and symptomatic) confirmed by positive parasitaemia on a blood slide; incidence of grade 3 or 4 adverse events; mean change in CD4 and haematologic indices after 12 months; adherence to use of ART, trial drug and ITN.

### 2.8 Assessment of adherence

Adherence to the use of trial drug, ART and ITN is assessed at every scheduled and unscheduled visit using a standard adherence questionnaire and by returned trial drug and ART pill counts. Adherence counselling is given at every visit. An exit interview questionnaire will be administered to capture the possible ingestion of CTX either from supplies left over prior to enrolment or from sources outside the trial during follow-up.

### 2.9 Sample size

Sample size calculations used the following assumptions: the rate of CTX preventable events in the control arm would be 10 per 100 PYO, based on an analysis of event rates from the DART trial [9] among participants with confirmed CD4 count above 250 cells/mm^3^; loss to follow-up rate would be 4% per year; type I error of 0.05 (one-sided for non-inferiority), the upper limit of the 95% confidence interval for the hazard ratio comparing the intervention arm with the control arm should be at most 1.25 in order to demonstrate non-inferiority; power of 80% assuming equal event rates in the two arms. Under these assumptions a total of 2000 participants would be required, among whom a total of 494 CTX preventable events would be expected. For the co-primary safety end-point a sample size of 1000 per arm would have over 80% power to detect a halving in the rate of grade 3 or 4 haematological events at the 5% level, assuming that at least 10% of those in the arm that continues to receive CTX prophylaxis experience such an event. The sample size was estimated using the formula of Schoenfeld [10].

### 2.10 Trial oversight

The overall trial oversight is provided by the Trial Steering Committee (TSC). There is an unblinded Independent Data Monitoring Committee (IDMC) that meets six monthly and is responsible for reviewing study recruitment targets, the safety and efficacy endpoints and the available external evidence from other related studies. The IDMC is also responsible for advising the TSC on whether to stop, amend or continue the trial as originally planned. The IDMC can recommend stopping the trial if there is overwhelming evidence (as determined by the Peto–Haybittle rule) [11,12] of a difference in the rate of CTX-preventable events between the two arms.

### 2.11 Ascertainment of the primary endpoints

All potential primary endpoints are captured by the study clinicians and adjudicated by the ERC. Haematological events (anaemia, neutropenia and thrombocytopenia) are assessed through scheduled laboratory tests carried out in the MRC laboratories. Critical CD4 count measurements (< 250 cells/mm^3^) are confirmed with a repeat test. The DAIDS toxicity grading tables [13] are utilized to grade the severity of the measured laboratory parameters.

### 2.12 Data management and quality assurance

A database for the COSTOP trial is custom designed in MS ACCESS. All CRFs are printed in duplicate and the data is double-entered and validated before being uploaded into the database. The data from the Masaka site is transferred and merged with the main trial database in Entebbe every two weeks. The monitoring of the trial to assess adherence to the protocol, respect of participant rights and data quality is done routinely by the MRC/UVRI Uganda Research Unit on AIDS monitors and monitors from the East African Consortium for Clinical Research (EACCR) and these are blinded to the treatment allocation.

### 2.13 Analysis plan

Two data sets will be used for analysis namely per protocol (PP) and intention to treat (ITT) populations. The PP population will consist of all subjects who were considered to have taken at least 80% of their blinded study medication in each period between scheduled study visits. Study participants will remain in the per protocol population as long as their adherence as defined above remains at 80% or higher, and will be dropped from the PP data set at the visit at which their adherence during that period drops to below 80%. Such patients will not re-enter the PP data set. The ITT data set will consist of all subjects who took at least one dose of blinded study medication and for whom there is at least one follow-up assessment.

For the primary analysis of the first co-primary endpoint, namely time to first CTX preventable event or death, the analysis will test for non-inferiority (NI), hence the main analysis will be a per-protocol (PP) analysis. An ITT analysis will also be done on this population as a form of sensitivity analysis. For the primary analysis of the second co-primary endpoint (time to first haematological grade 3 or 4 adverse event) and for all secondary endpoints, analysis will be carried out on the ITT population.

For all time to event analyses, a subject will be considered to be part of the trial until the subject experiences the event, or the trial ends, the subject leaves the trial (due to withdrawal or loss to follow-up), the subject dies or in the case of the co-primary non-inferiority endpoint, the subject no longer qualifies for the per protocol population.

The comparative incidence of first clinical events in the two study arms will be illustrated graphically using Kaplan Meier plots. The incidence rate in each arm will be estimated together with 95% confidence limits, since it is considered safe to stop CTX-prophylaxis if the event rate in the experimental arm is sufficiently low (upper limit of 95% confidence interval is below 1 per 100 pyar), even if the formal non-inferiority limit is not met. Non-inferiority will be tested by fitting a Cox proportional hazards regression model with terms for centre (Entebbe or Masaka), CD4 stratum (250–499 cells/mm^3^ *vs.* 500 or more cells/mm^3^) and treatment arm and calculating the one sided 95% confidence limit for the hazard ratio for the experimental arm (stopping CTX) relative to the control arm (continuing CTX). The experimental arm will be deemed to be non-inferior to the control arm if the upper limit of the confidence interval is less than 1.25, that is, no more than a 25% increased risk of an endpoint event on the placebo arm. In investigating whether stopping CTX prophylaxis is superior to continuing CTX prophylaxis with respect to the safety endpoint of time to the first grade 3 or grade 4 haematological adverse events, an intention-to-treat approach will be used.

The frequency of such events will be tabulated by treatment arm, separately for neutropenia, anaemia and thrombocytopenia. Further analyses will be carried out using survival analysis methods. The incidence of grade 3 or grade 4 haematological adverse events will be illustrated graphically using Kaplan Meier plots. The primary analysis will be carried out by means of a log-rank test, stratified by the four randomization strata defined by the combinations of study site and CD4 stratum, to compare the event rates between the two study arms. Further analysis will be carried out by fitting a Cox proportional hazard regression model, with terms for site, CD4 stratum and treatment arm.

## 3 Discussion

The aim of COSTOP is to assess whether CTX prophylaxis can be safely discontinued among HIV-infected African adults that have achieved sustained immune reconstitution following initiation of ART in resource limited settings. According to the recent WHO guidelines [2], completed studies that have evaluated this concept have provided moderate to low quality evidence and the main reason for this is related in part to their design.

Most clinical trials involving the use of drugs are ‘forward sighted’, comparing a new drug to a standard, placebo or no treatment at all. In a situation where stopping the standard treatment (intervention) needs to be compared to the standard treatment itself (control), the choice of study design and the methodological considerations to be made can be quite challenging. A clear example in this case is the design and conduct of CTX cessation studies.

In our study a non-inferiority design was required since the goal was to determine whether withdrawing CTX would not disadvantage the participant. The use of a matching placebo in the intervention arm in which CTX is withdrawn is particularly important to avoid possible biases associated with knowledge of whether the patient is receiving prophylaxis. It would be very difficult to maintain cessation of CTX prophylaxis among patients randomised to do so in a setting where CTX is readily and cheaply available and CTX widely believed to be always beneficial. Adherence of participants to their allocated treatment arm is clearly much easier if they are unaware of whether they are receiving CTX or placebo. If either the investigators or the patients feel that those allocated to placebo are disadvantaged, this may lead to differential reporting of events and even premature withdrawal of patients from the trial. This could lead to failure to demonstrate non inferiority when it actually exists (type II error). Without double blinding, both the patients and investigators may perceive this to be true based on the previous evidence that HIV infected patients on ART who stop CTX may be at a higher risk of experiencing HIV-related mortality and morbidity [7] while those continuing on CTX may be at risk of adverse drug reactions.

The dose of CTX is maintained at 960 mg daily as is the current practice since a reduction could result in increased event rates in the control arm making it no longer a true control. Study drug and ART adherence are strictly monitored as differential rates of adherence could lead to erroneous conclusions [14]. The primary outcome is a composite of morbidity and mortality due to all CTX preventable WHO staging events. The criteria developed by WHO for the diagnosis of these events is based on both definitive and presumptive evidence by the clinician. In this study, an Endpoint Review Committee is established to ensure precise ascertainment of endpoints based on the presumptive/definitive WHO criteria [15]. Since some diagnoses based on this criterion involve some degree of subjectivity, in the absence of the ERC, a high proportion of incorrect diagnoses could add “noise” to the trial results and hence diminish the difference in the arms. All these design considerations are necessary for the non-inferiority trial to be conducted with rigour and avoid making false conclusions about the effectiveness of the intervention [16].

We are aware of concerns that patients might have access to unused supplies of CTX or be able to purchase CTX during the study. Ideally, adherence to trial medication would be best assessed by serum level measurements of CTX or its metabolites. However, there is no suitable method that would only detect serum or urine levels of CTX or its metabolites without being affected by other drugs that patients take. In the absence of a suitable test it was decided to conduct end-of-trial interviews by independent researchers in order to identify patients who had possibly taken open label CTX at some time during the trial and had correctly reported this to the trial team. If participants have such access, non-inferiority might be inappropriately demonstrated.

In conclusion, this paper describes the design and methods used in the COSTOP trial highlighting some important aspects in designing CTX cessation studies. It is expected that the design and methodological considerations will significantly contribute to the quality of evidence from this study.

## Conflict of interest

No conflict of interests declared by the authors.

## Authors' contributions

PM, JL, RK and HG conceived the initial trial idea. ZA, AA, AN and JL prepared the initial draft of this manuscript. RK, AK, HG and PM reviewed and made substantive contributions to subsequent drafts. All authors have read and approved the final manuscript.

## Figures and Tables

**Table 1:** Cotrimoxazole preventable WHO staging events.

Cotrimoxazole preventable events
*WHO clinical stage 4*
Pneumocystis pneumonia
Recurrent severe bacterial pneumonia
Central nervous system toxoplasmosis
Chronic isosporiasis
Recurrent non-typhoidal salmonella bacteraemia

*WHO clinical stage 3*
Unexplained severe weight loss (> 10% of presumed or measured body weight)
Unexplained chronic diarrhoea for longer than one month
Unexplained persistent fever (above 37.6 °C intermittent or constant, for longer than one month)
Severe bacterial infections (such as pneumonia, empyema, pyomyositis, bone or joint infection, meningitis or bacteraemia)
Acute necrotizing ulcerative stomatitis, gingivitis or periodontitis
Unexplained anaemia (< 8 g/dl), neutropaenia (< 0.5 × 10^9^ per litre)
or chronic thrombocytopaenia (< 50 × 10^9^ per litre)

*WHO clinical stage 2*
Moderate unexplained weight loss (< 10% of presumed or measured body weight)
Recurrent respiratory tract infections (sinusitis, tonsillitis, otitis media and pharyngitis)

**Table 2:** Summary of study schedule of visits and procedures.

Procedure	Assessment time							
	Screening (weeks − 2 to − 4)	Enrolment (week 0)	Week 4	Week 8	Week 12	Week 24	Every 3 months after	Every 6 months after
Consent for screening	x							
Consent for plasma storage		x						
Consent for enrolment		x						
History & physical examination^a^	x	x	x	x	x	x	x	
CD4 count^b^	x	x			x	x		x
Full blood count	x				x	x	x	
Pregnancy test^c^	x							
Malaria slide		x	x	x	x	x	x	
Adherence assessment		x	x	x	x	x	x	
Study drug prescription/refill		x	x	x	x	x	x	

a Doctor assessment. Including a record of all clinical events and any adverse events since previous visit.

b CD4 counts at baseline, after first three months, after 6 months and then 6 monthly thereafter.

c Pregnancy test in all women of reproductive age at screening. Thereafter, only in event of amenorrhea.
